# Editorial: Emerging antimicrobials: sources, mechanisms of action, spectrum of activity, combination antimicrobial therapy, and resistance mechanisms

**DOI:** 10.3389/fmicb.2025.1754398

**Published:** 2025-12-16

**Authors:** Piotr Majewski, Amanda Claire Brown, Thamarai Schneiders, John Osei Sekyere

**Affiliations:** 1 Medical University of Bialystok, Białystok, Poland; 2 Tarleton State University, Stephenville, TX, United States; 3 The University of Edinburgh, Edinburgh, United Kingdom; 4 Genesis Biotechnology Group LLC, Trenton, NJ, United States; 5 Department of Medical Microbiology, School of Medicine, University of Pretoria, Prinshof Campus Pretoria, South Africa

**Keywords:** antimicrobial resistance, MDR, XDR, Gram-negative bacteria, tuberculous mycobacteria, emerging fungal pathogens, *Candida auris*, novel natural products

## Urgent need for innovative antimicrobials

Antimicrobial resistance (AMR) has escalated into a global health crisis, with multidrug-resistant (MDR) and even pan- or total-drug-resistant pathogens rendering once-curable infections difficult or impossible to treat. In the United States alone, invasive infections by MDR and extensively drug-resistant (XDR) bacteria cause an estimated 2.8 million infections and 35,000 deaths annually. Mortality rates for invasive MDR infections can exceed 50% globally, and the economic burden is staggering, e.g., over $4.6 billion in U.S. healthcare costs per year (CDC. Antibiotic Resistance Threats in the United States, 2019) (O'Neill J. Tackling drug-resistant infections globally: final report and recommendations. Review on Antimicrobial Resistance). Without effective antibiotics, routine medical procedures, from pneumonia treatment to surgical prophylaxis and chemotherapy, become high-risk, raising the prospect of a “post-antibiotic era” in which common infections may once again become untreatable. This pressing threat of AMR motivates urgent exploration of new antimicrobials and strategies.

However, most antibiotics in use today are variations of a handful of drug classes discovered in the mid-20th century, often derived from soil actinomycetes. This pipeline has largely been exhausted. Pathogens have evolved resistance to nearly all available drugs, outpacing our drug development. Notably, critical gaps exist for hard-to-treat organisms: certain Gram-negative bacteria, tuberculous mycobacteria, and emerging fungal pathogens now exhibit MDR or even pan-resistance. The antifungal arsenal is similarly limited, with lethal yeasts like *Candida auris* and mold developing drug resistance. To avert a public health catastrophe, the scientific community is striving to discover antimicrobials with truly novel targets and modes of action, to revive older compounds through innovative approaches, and to optimize combination therapies capable of outmaneuvering contemporary resistance mechanisms.

In this Research Topic, the contributing authors addressed this challenge by examining a broad spectrum of solutions, from novel natural products and antimicrobial peptides to drug repurposing, synergistic combinations, and deeper investigations into microbial strategies of drug evasion. Spanning both bacteria and fungi, and encompassing experimental studies, clinical observations, and comprehensive reviews, these articles collectively underscore the multi-pronged approach required to combat AMR. In this editorial, we synthesize the key findings and thematic threads emerging from these contributions, highlighting how they advance the pursuit of effective new antimicrobial strategies.

## Diverse sources of novel antimicrobials

A recurring theme is the search for new antimicrobial agents from diverse and even unconventional sources. Several works in this Research Topic underscore that promising compounds can be found in nature's reservoirs, if we look in the right places.

Actinomycetes (Gram-positive filamentous bacteria inhabiting soil and other ecological niches) have historically yielded the majority of clinically used antibiotics. As classical soil-derived strains have been extensively mined, attention has increasingly shifted toward underexplored environments. In this context, Helmi provides a comprehensive systematic review of actinomycetes isolated from Saudi Arabia's diverse ecosystems, including desert soils, rhizospheres, caves, and mangroves. The review highlights that the extreme conditions characteristic of these habitats have selected for unique actinomycete lineages with enhanced biosynthetic potential for bioactive secondary metabolites. Notably, Saudi isolates produce compounds active against major MDR pathogens such as methicillin-resistant *Staphylococcus aureus* (MRSA), ESBL-producing *Enterobacterales, Pseudomonas aeruginosa*, and even *Candida* species. These findings underscore the capacity of extremophilic and rare actinomycetes to serve as rich sources of novel antimicrobial scaffolds. The review also emphasizes the role of emerging technologies, genome mining, metagenomics, AI-driven analyses, and synthetic biology in unlocking cryptic biosynthetic gene clusters and improving compound production. While the primary focus is antimicrobial discovery, Helmi also notes that actinomycete-derived metabolites possess broader biomedical relevance, including antitumor activities, echoing observations by Rui et al.

Shifting from microbial sources to host-derived molecules, Chatterjee and Sivashanmugam examine antimicrobial peptides (AMPs) as promising therapeutic alternatives. Their review of immunomodulatory peptides spans a wide range of host-defense peptides identified in humans, animals, plants, and amphibians. These AMPs not only exert direct antibacterial, antifungal, and antiviral activity but also modulate host immunity, for example, by recruiting immune cells or tempering excessive inflammation. The authors catalog numerous peptide families, from human defensins and cathelicidins to amphibian-derived magainins, and discuss their mechanisms of action and therapeutic applications. By acting simultaneously on microbial membranes and host immune pathways, AMPs offer a dual antimicrobial-immunomodulatory strategy that may help counter MDR infections. This revitalizes interest in biologically inspired therapeutics capable of circumventing conventional resistance mechanisms. Notably, many AMPs target bacterial membranes or intracellular components through mechanisms distinct from classical antibiotics, making it difficult for pathogens to develop resistance. Harnessing or optimizing these natural peptides, and their synthetic derivatives could substantially expand the antimicrobial arsenal with genuinely new classes of agents.

Nature also provides a wealth of phytochemicals and essential-oil constituents with antimicrobial activity, forming a promising basis for plant-derived therapeutics (Li et al.). Two original research articles in this Research Topic evaluate such compounds against challenging fungal pathogens. Li et al. investigate cinnamaldehyde, the major bioactive component of cinnamon bark, for activity against *Aspergillus fumigatus*, a mold responsible for invasive aspergillosis. They report potent fungistatic effects and show that cinnamaldehyde disrupts the tricarboxylic acid (TCA) cycle and protein metabolism in *A. fumigatus*. Metabolomic and proteomic analyses reveal down-regulation of key TCA-cycle enzymes and amino-acid metabolic pathways, impairing fungal energy production and growth. This metabolic targeting differs markedly from the mechanisms of azoles and polyenes, suggesting that cinnamaldehyde and related analogs may overcome resistance by acting on novel biochemical pathways. In a complementary study, Wang et al. examine terpinen-4-ol, a constituent of tea tree oil, against *Botrytis cinerea*. Although *B. cinerea* is primarily a plant pathogen, it is widely used as a model for dissecting antifungal mechanisms. The authors demonstrate that terpinen-4-ol induces an autophagy-like programmed cell death involving activation of fungal autophagy pathways and a metacaspase-dependent apoptotic process. In essence, the compound triggers self-destructive responses within the fungal cell. Together, these studies underscore the substantial potential of plant-derived antimicrobials: by disrupting fundamental metabolic or regulatory processes, such compounds may yield new antifungal or antibacterial agents. Importantly, their distinct mechanisms of action could retain efficacy against strains resistant to existing drugs.

## Repurposing and innovative therapeutic strategies

Beyond the discovery of entirely new molecules, drug repurposing offers an efficient strategy to expand the antimicrobial toolbox by identifying antibacterial activity in existing drugs or biochemical compounds not originally developed as antibiotics. Several contributions in this Research Topic illustrate how repurposed agents and alternative anti-infective approaches, often aimed at virulence or survival pathways rather than direct killing, can complement traditional therapeutics. A striking example is disulfiram, an alcohol-aversion drug now shown to possess unexpected antibacterial properties. Luo et al. report that disulfiram inhibits the growth of both Gram-positive and Gram-negative bacteria by inducing intracellular oxidative stress. Their mechanistic analyses demonstrate that disulfiram is bacteriostatic and triggers substantial accumulation of reactive oxygen species (ROS). Notably, this activity depends on intracellular zinc: zinc chelation or deletion of zinc-import systems in *E. coli* mitigated disulfiram's effects, whereas zinc supplementation enhanced them. The compound appears to disrupt metal homeostasis and redox balance, weakening antioxidant defenses and compromising membrane integrity. Although disulfiram alone did not rapidly kill bacteria, a key finding was its strong synergy with bactericidal agents such as colistin and kanamycin. In a murine infection model, combining disulfiram with colistin improved *E. coli* clearance relative to monotherapy. These results position disulfiram as a potential antibiotic adjuvant, sensitizing pathogens through redox destabilization and thereby enhancing the activity of established drugs. While standard human dosing may not achieve bacteriostatic plasma concentrations, its repurposing potential remains compelling, particularly given its FDA approval, oral availability, and long clinical safety record. Luo et al.'s study also highlights dithiocarbamates, the chemical class of disulfiram, as promising scaffolds for future antimicrobial or adjuvant development.

Another repurposed agent examined in this Research Topic is 5-fluorouracil (5-FU), a well-established chemotherapeutic antimetabolite. Niazy et al. present a Mini Review on the effects of 5-FU on *P. aeruginosa*, with an emphasis on virulence and biofilm formation. *P. aeruginosa* is a notorious MDR pathogen characterized by its formidable capacity to form biofilms in chronic infections. As an uracil analog, 5-FU can interfere with bacterial nucleotide metabolism. The review highlights that sub-inhibitory concentrations of 5-FU markedly suppress biofilm development and the production of quorum-sensing–regulated virulence factors. Recent studies demonstrate reduced levels of key virulence determinants, such as pyocyanin and proteases, as well as impaired biofilm maturation, often without substantial effects on planktonic growth at these doses. By targeting bacterial communication and virulence rather than directly inhibiting growth, 5-FU represents a promising anti-virulence strategy that may facilitate host clearance or enhance antibiotic activity. Such approaches are appealing because they impose comparatively low selective pressure for resistance: the pathogen is disarmed rather than killed. The authors discuss how 5-FU and related antimetabolites could be repurposed as adjunctive therapies in difficult-to-treat infections where biofilms and virulence drive pathology. An approved drug like 5-FU, if appropriately formulated for localized delivery, could therefore find renewed utility as an anti-infective agent that attenuates pathogenicity.

Repurposing efforts also extend to antiparasitic agents. Yuan et al. investigate naphthoquine phosphate, an antimalarial compound for activity against *Acinetobacter baumannii*, a critical-priority MDR pathogen. Given the widespread resistance of *A. baumannii*, particularly carbapenem-resistant strains, any new active agent is of considerable interest. Strikingly, the authors report potent antibacterial activity of naphthoquine phosphate against a ceftazidime-resistant *A. baumannii* isolate. Mode-of-action studies indicate that the compound disrupts bacterial membrane integrity while simultaneously inducing the accumulation of ROS. This dual action, physical membrane damage coupled with oxidative stress, ultimately leads to cell death, reminiscent of certain aspects of polymyxin activity combined with intracellular stress induction. The finding that an antimalarial drug can effectively kill a Gram-negative superbug exemplifies the potential of cross-domain repurposing. It underscores the value of examining drug libraries developed for parasitic, oncologic, or other non-bacterial diseases for hidden antimicrobial activity. With further optimization, naphthoquine or related derivatives could form the basis of a new antibiotic scaffold, benefiting from the fact that some human safety data already exist from its antimalarial use.

In addition to conventional drugs, non-traditional antimicrobials such as prebiotic sugars are also being explored. Asbury and Saville investigate β-manno-oligosaccharides (MOS), mannan-derived carbohydrates as a novel anti-infective approach. MOS are not inherently bactericidal, but they can inhibit early infection by preventing bacterial adhesion to host tissues. In their study, multiple MOS preparations were tested against *E. coli, Klebsiella pneumoniae, Listeria monocytogenes*, and *Streptococcus mutans*. Although MOS alone exerted species-specific growth-inhibition effects, a key finding was their pronounced ability to potentiate certain antibiotics. For example, MOS markedly enhanced the efficacy of ceftazidime against *K. pneumoniae*. The authors propose that MOS may bind to bacterial surfaces or biofilm matrices, increasing permeability or otherwise facilitating antibiotic entry, analogous in concept to adjuvants such as disulfiram or polymyxins, but achieved through a benign sugar. As a non-toxic prebiotic already used in feed applications, MOS could represent a safe adjunct to antibiotic therapy, improving outcomes or reducing required doses. This aligns with the broader trend toward anti-adhesion and anti-virulence strategies: rather than killing pathogens directly, MOS block the initial attachment step and, in doing so, render bacteria more susceptible to antimicrobial action.

## Enhancing efficacy through combination therapy

Combination antimicrobial therapy remains a cornerstone of contemporary strategies to counter resistance. Using drugs in concert can yield synergistic killing, expand antimicrobial coverage, and suppress the emergence of resistant mutants. Several contributions in this Research Topic focus on such combination approaches, ranging from dual-antibiotic regimens to antibiotic-adjuvant pairings, and on the methodological tools that enable their rational design.

A comprehensive review by Ramirez and Schweizer addresses a central obstacle in treating Gram-negative infections: the impermeable outer membrane. This lipid-rich barrier prevents many antibiotics from reaching their intracellular targets. Overcoming it can restore the activity of drugs that are otherwise ineffective against Gram-negative pathogens. The authors summarize recent efforts to develop outer-membrane permeabilizers derived from polymyxins and aminoglycosides. Polymyxins, such as colistin, naturally bind to LPS and disrupt membrane integrity, but their nephrotoxicity limits therapeutic use. Current research therefore focuses on generating non-antibiotic polymyxin derivatives that retain membrane-targeting properties while minimizing toxicity, agents intended solely as adjuvants to facilitate antibiotic entry. Similarly, certain aminoglycosides can induce self-promoted uptake through membrane disruption; chemists are modifying these molecules to enhance permeabilization without relying on their ribosome-targeting activity. The review highlights how such “helper” compounds can dramatically potentiate antibiotics that typically show poor activity against Gram-negatives, for example, enabling rifampicin or vancomycin to become effective when paired with a permeabilizer. This underscores a broader concept recurring across the Research Topic: antibiotic adjuvants that exhibit modest intrinsic activity but substantially amplify the potency of true antibiotics. By weakening the protective outer membrane, permeabilizing adjuvants can effectively circumvent intrinsic resistance and revive older antibiotic classes for modern use. It is a strategically elegant approach: dismantle the pathogen's shield so the antibiotic can reach, and act on its target.

Combination therapy can also involve pairing two potent antibiotics to manage severe infections. Yet clinical evidence supporting newer combinations remains relatively scarce. In this Research Topic, Chen, Chen, Hu et al. contribute a matched-cohort clinical study evaluating intravenous omadacycline, a modern tetracycline derivative, for the treatment of severe pneumonia caused by carbapenem-resistant *A. baumannii* (CRAB). CRAB pneumonia is associated with extremely high mortality and very limited therapeutic options, with colistin and tigecycline often serving as last-resort agents. Using propensity-score matching, the authors compared outcomes in patients treated with omadacycline vs. those receiving tigecycline, the current standard of care. Their pilot investigation, the first of its kind, found that omadacycline achieved clinical efficacy comparable to tigecycline for CRAB pneumonia. Importantly, omadacycline was linked to fewer adverse effects, in contrast to tigecycline's well-known nausea and hepatotoxicity profiles, suggesting a potential tolerability advantage. These preliminary clinical findings are encouraging: they indicate that omadacycline, initially approved for community-acquired pneumonia and skin infections, may be repurposed or expanded for the management of highly resistant hospital pathogens. In practice, omadacycline could be incorporated into combination regimens for CRAB. This study not only opens the door to larger clinical trials but also underscores the value of real-world evidence in guiding antibiotic use in challenging, high-resistance settings.

In some cases, the tools that enable combination therapy constitute the core contribution. Wülbern et al. present a *Methods* article describing an approach to assess the antibiotic susceptibility of predatory bacteria. Predatory species such as *Bdellovibrio bacteriovorus*, which prey on Gram-negative pathogens, are being investigated as living antimicrobials. To employ such organisms therapeutically (for example, to target *P. aeruginosa* in pulmonary infection), it is essential to determine which antibiotics can be co-administered without impairing the predator. The authors developed a streamlined assay that measures how predatory bacteria respond to antibiotics while they form plaques on prey lawns. This methodological advance is fundamental for designing combination biotherapies, ensuring that predatory bacteria, phages, and conventional antibiotics can be used together safely and effectively. It reflects forward-looking preparation for a therapeutic landscape in which cocktails of antibiotics, phages, probiotics, and other biological agents may be deployed in tandem. Robust methods to evaluate compatibility and susceptibility of each component will be indispensable for such integrated antimicrobial strategies.

Finally, combination strategies extend beyond antibacterials themselves. A holistic response to AMR requires integrating multiple therapeutic modalities. In their wide-ranging review, Karnwal et al. discuss non-traditional adjuncts, including bacteriophages, probiotics, and immunotherapies, alongside antibiotics. They highlight how combining antibiotics with phage therapy can enhance bacterial clearance while reducing the likelihood of resistance development. Likewise, probiotic interventions or targeted dietary modifications may help reshape the microbiome to lower the prevalence of resistance genes. The overarching theme is that no single intervention will suffice to counter AMR. Instead, effective control will rely on integrated approaches that pair antibiotics with biological agents and host-directed strategies such as monoclonal antibodies, vaccines, or nanoparticle-based therapeutics. This reflects an expanded view of combination therapy: not only antibiotic-antibiotic pairings, but coordinated regimens of antibiotics, phages, immune modulators, and microbiome-based interventions acting synergistically against resistant pathogens.

## Emerging resistance and surveillance insights

Understanding *how* and *where* resistance emerges is as critical as developing new treatments. Several studies in this Research Topic investigate resistance mechanisms and epidemiological trends, providing insights that can inform the strategic use of emerging antimicrobials.

On the molecular front, Chen, Chen, Zhang et al. investigate the relationship between antibiotic resistance and virulence in *Klebsiella pneumoniae*, comparing carbapenem-resistant and carbapenem-susceptible strains. Their analysis reveals that carbapenem-resistant *K. pneumoniae* (CRKP) isolates, despite their high-level drug resistance, tend to carry fewer classical virulence determinants, whereas susceptible strains retain a broader array of virulence genes. Under antibiotic pressure *in vitro*, susceptible strains upregulated key virulence regulators—such as the capsule-associated genes *rmpA/A2*—while resistant strains instead increased expression of resistance genes, including *bla*_KPC_ and *bla*_SHV_. These findings suggest an adaptive trade-off: *K. pneumoniae* may differentially allocate resources toward virulence or resistance depending on environmental pressures. CRKP isolates, already burdened with resistance determinants, may downregulate energetically costly virulence traits to enhance survival under antimicrobial stress, whereas susceptible strains lacking robust resistance mechanisms may maintain, or even enhance virulence potential. This “intricate relationship” between virulence and resistance has clear clinical implications. CRKP strains driving hospital outbreaks may be less hypervirulent than community-associated lineages, yet their profound resilience to antibiotics renders them highly problematic. The study underscores the importance of surveillance systems that track both resistance genotypes and virulence phenotypes. Moreover, as several contributions in this Research Topic focus on anti-virulence strategies, understanding how pathogens balance these traits is essential for predicting evolutionary responses to new therapeutic interventions.

Another contribution, by Addis et al., offers a snapshot of *Staphylococcus aureus* resistance patterns in Ethiopia. In this cross-sectional clinical study, *S. aureus* was isolated from 11.2% of patients with suspected bacterial infections; notably, more than one-third of these isolates were MRSA, and over one-quarter exhibited inducible clindamycin resistance. The latter finding indicates a substantial prevalence of *erm*-mediated inducible MLS_B_ resistance, detectable via the D-test. The authors also identified socioeconomic and demographic factors associated with increased risk of *S. aureus* infection and resistance, such as larger household size and lower income, potentially reflecting transmission dynamics and disparities in healthcare access. The presence of multidrug-resistant *S. aureus* in this regional context underscores the global pervasiveness of MRSA and highlights the urgent need for new anti-staphylococcal agents, including both next-generation anti-MRSA antibiotics and anti-virulence approaches. Importantly, the study illustrates how local epidemiology shapes therapeutic decision-making. Developers of emerging antimicrobials must consider region-specific resistance mechanisms: for instance, high rates of inducible macrolide-lincosamide resistance may limit the utility of clindamycin-based regimens in Ethiopia, whereas agents capable of bypassing *erm*-mediated resistance could provide significant benefit.

Fungal pathogens are also undergoing rapid evolution. Ibe and Pohl contribute a thought-provoking Mini Review proposing that climate change and thermal adaptation may be key drivers of emerging multidrug-resistant fungi. They argue that rising global temperatures, and the corresponding need for environmental fungi to withstand mammalian body temperature, may select for strains with cross-resistance to antifungal drugs. The enigmatic near-simultaneous emergence of *C. auris* on three continents is cited as a prominent example: one hypothesis suggests that environmental warming forced *C. auris* and related species to adapt to higher temperatures, activating stress-response pathways that incidentally conferred drug resistance. The authors note that many newly emerging fungal pathogens, including *C. auris*, are already multidrug- or even pan-drug-resistant at the time they are first recognized in humans. This pattern implies that resistance may arise in environmental settings before clinical emergence, potentially as a byproduct of surviving thermal or ecological stress. They highlight how heat-induced cellular changes, such as upregulation of heat-shock proteins and other stress-response regulators, can activate antifungal resistance mechanisms even in the absence of drug exposure. This provocative link between climate change and antifungal resistance broadens the AMR discourse: surveillance must extend beyond clinical environments to include environmental reservoirs. Resistance is not driven solely by hospital antifungal use; warming climates and ecological pressures may generate resistant fungi in nature that subsequently enter healthcare settings. Ibe and Pohl call for intensified monitoring of environmental fungi and deeper investigation into how thermal adaptation may pre-adapt pathogens to resist current antifungal therapies. Their perspective aligns with the One Health framework, emphasizing the interconnectedness of human, animal, and environmental health in the resistance crisis.

Lastly, Deng et al. provide a 5-year multicentre surveillance study of bloodstream fungal infections in Sichuan, China, offering a macro-level view of regional antifungal resistance trends. Across more than 2,000 fungemia cases collected between 2019 and 2023, *Candida* species accounted for nearly 89% of isolates, with *Candida albicans* remaining the predominant species (38%). Reassuringly, *C. albicans* retained high susceptibility to fluconazole (~91%), and both *Candida parapsilosis* and wild-type *Candida glabrata* showed >80% susceptibility to voriconazole. However, the dataset also revealed concerning resistance patterns. *Candida tropicalis* isolates exhibited high resistance rates to fluconazole (36%) and voriconazole (35%), reinforcing its emergence as a resistant pathogen in parts of Asia. *Cryptococcus neoformans* isolates demonstrated non-wild-type (reduced susceptibility) rates of approximately 5–9% across major antifungals. The study further noted hospital-type variation, for example, pediatric hospitals showed a higher proportion of *C. parapsilosis* fungemia, consistent with its known association with neonatal units. Deng et al. conclude that significant regional variability in species distribution and antifungal susceptibility underscores the importance of localized epidemiological data to guide therapy. Such surveillance programs are essential for detecting shifts in pathogen prevalence and resistance. For developers of new antifungals, these data are highly informative: given the high azole resistance of *C. tropicalis*, agents with strong activity against non-albicans *Candida*, and ideally also against pan-resistant *C. auris* would address critical clinical gaps. Although *C. auris* was not detected in this dataset, its global rise serves as a reminder that future antifungal agents must be designed with these emerging threats in mind.

## Toward a multidimensional response to AMR

Taken together, the articles in this Research Topic present a picture that is both encouraging and complex. Scientific innovation is steadily expanding the antimicrobial arsenal: from unusual environmental microbes yielding novel antibiotics, to repurposed drugs revealing unexpected activities, to synergistic combinations and therapeutic strategies that attenuate virulence or enhance host defenses. These contributions illustrate that antimicrobials can originate from a remarkable diversity of sources, soil actinomycetes, marine sediments, medicinal plants, FDA-approved drug libraries, and even host-derived peptides. At the same time, the spectrum of action is broadening. Researchers are developing agents capable of breaching the formidable outer membrane of Gram-negative bacteria, as well as adjuvants that disable resistance mechanisms and restore the activity of existing drugs. A consistent theme across the Research Topic is the primacy of combination approaches: each study adds an element to an emerging multi-pronged therapeutic paradigm, for example, antibiotic plus adjuvant plus immune modulation, rather than relying on a single molecule to solve the resistance crisis. Collectively, these advances reflect a maturation of the field: a shift away from the traditional one-drug-one-pathogen mindset toward more integrated, combinatorial, and systems-level strategies to overcome AMR.

On the other hand, microbes are far from static targets. They adapt continuously through horizontal gene transfer, metabolic reprogramming, and as emerging evidence suggests, even climate-driven evolutionary pressure. Surveillance and mechanistic studies in this Research Topic underscore that resistance can arise from unexpected directions. A single point mutation or mobile genetic element can compromise a frontline antibiotic, and shifts in microbial behavior, such as biofilm formation or entry into dormancy can render otherwise potent drugs ineffective. Consequently, detailed knowledge of resistance pathways and epidemiological trends must guide both drug development and clinical deployment. Several authors emphasize that prudent, context-specific use of new therapeutics is essential. For instance, indiscriminate introduction of a novel antifungal without understanding local species distributions and susceptibilities may undermine its effectiveness; aligning interventions with pathogen profiles, as highlighted by Deng et al., maximizes clinical success. Likewise, Karnwal et al. remind us that scientific advances must be accompanied by robust stewardship and policy measures. New antimicrobials alone cannot overcome AMR if misuse persists, an undercurrent running throughout this Research Topic, even when not the primary focus of individual studies.

In conclusion, the contributions gathered in this Research Topic exemplify the remarkable breadth of contemporary antimicrobial research. They span the continuum from bench to bedside and from molecular discovery to population-level surveillance, embodying the multifaceted approach required to confront AMR. Equally important, they highlight the value of cross-disciplinary collaboration, microbiologists, chemists, clinicians, and epidemiologists each advancing critical facets of the field. Such integrated efforts offer our best prospect for averting a post-antibiotic future and instead fostering a new era of antimicrobial innovation and responsible use.

As Topic Editors, we extend our sincere appreciation to all contributing authors for sharing their research work and to the expert reviewers for their thoughtful evaluations. We hope readers will find both inspiration and practical insights within these articles. By linking novel compounds, unconventional mechanisms, combination strategies, and resistance surveillance, this Research Topic brings us a step closer to understanding drug-resistant infections. The challenge is urgent, but the collective ingenuity demonstrated here charts a promising course toward sustainable control of infectious diseases in the 21st century.

